# Infections with *Giardia duodenalis* and *Entamoeba histolytica*/*Entamoeba dispar* as Hidden and Prevalent Conditions in Periurban Communities in the State of Rio de Janeiro, Brazil

**DOI:** 10.1155/2020/3134849

**Published:** 2020-07-14

**Authors:** Deiviane A. Calegar, Kerla J. L. Monteiro, Andressa B. Gonçalves, Márcio N. Boia, Lauren H. Jaeger, Beatriz C. Nunes, Filipe A. Carvalho-Costa

**Affiliations:** ^1^Fundação Oswaldo Cruz, Instituto Oswaldo Cruz, Laboratório de Epidemiologia e Sistemática Molecular, Rio de Janeiro RJ, Brazil; ^2^Escritório Técnico Regional-Fundação Oswaldo Cruz, Rua Magalhães Filho, 519, Centro/Norte, Teresina, Piauí, Brazil; ^3^Fundação Oswaldo Cruz, Instituto Oswaldo Cruz, Laboratório de Biologia e Parasitologia de Mamíferos Silvestres Reservatórios, Rio de Janeiro RJ, Brazil; ^4^Faculdade de Farmácia, Universidade Federal de Juiz de Fora, Rua José Lourenço Kelmer, S/n–Campus Universitário, Bairro São Pedro, Juiz de Fora, Minas Gerais, Brazil; ^5^Faculdade de Medicina de Petrópolis (FMP)/Faculdade Arthur Sá Earp Neto (FASE), Rua Machado Fagundes, 326, Cascatinha, Petrópolis, Rio de Janeiro, Brazil

## Abstract

This study aims to assess the prevalence, distribution, and etiological profile of intestinal parasitism in children living in periurban areas in Cachoeiras de Macacu, Rio de Janeiro, Brazil. A community-based cross-sectional survey (*n* = 479) was carried out. Prevalence of infection with *G. duodenalis* and *E. histolytica*/*E. dispar* was 8.6% (*n* = 41) and 13.4% (*n* = 64), respectively. Infection with *G. duodenalis* was significantly more frequent among children living in poor families (24/187 (12.8%) vs. 16/272 (5.9%); prevalence ratio (PR) = 2.18; 95% confidence interval (CI) = 1.19–3.99; *p*=0.011). This difference was also significant for infection with any pathogenic parasite (43/187 (23%) vs. 40/272 (14/7%); PR = 1.56; 95% CI = 1.06–2.30; *p*=0.026). In addition, people residing in houses with more than four inhabitants showed significantly higher positivity for infections with *G. duodenalis* and with *E. histolytica*/*E. dispar* (22/138 (15.9%) vs. 16/311 (5.1%); PR = 3.09; 95% CI = 1.68–5.71; *p* < 0.001 for *G. duodenalis* and 32/138 (23.2%) vs. 30/311 (9.6%); PR = 2.40; 95% CI = 1.52–3.79; *p* < 0.001 for *E. histolytica*/*E. dispar*). Laboratory diagnosis of protozoan enteric infections and effective drugs for their treatment are unmet goals in the primary health care system. Therefore, giardiasis and amebiasis are neglected conditions.

## 1. Introduction

Some species of protozoa with variable pathogenic potential inhabit the human intestine. *Giardia duodenalis* and *Entamoeba histolytica* are among those that are known to be harmful [1]. *G. duodenalis* negatively influences the development of children in a complex pathogenesis involving enterocyte apoptosis and immune-mediated reactions at the small intestine [2, 3]. Giardiasis usually not only presents an endemic epidemiological behaviour in developing countries but also causes outbreaks of diarrhoea in developed countries [4, 5]. Prevalence rates range from 10% to 30% in Brazil. In children hospitalized due to acute diarrhoea in Rio de Janeiro, the positivity rate was 4.7% [6, 7].

*E. histolytica* infections, despite being most frequently asymptomatic, have invasive potential and can be associated with dysentery and hepatic abscesses [8]. Amebiasis is also associated with poor water quality and sanitation, and its prevalence is substantially higher in developing countries [6]. In Brazil, prevalence rates of infection in nondiarrhoeal stools range from 12% to 25% in urban settings with poor sanitation infrastructure in Amazonian Brazil and 21% in semiarid regions under water stress in the northeast of the country. *E. histolytica* is indistinguishable, under light microscopy, from species considered to be nonpathogenic, for instance *Entamoeba dispar*. This leads to the use of the nomenclature *E. histolytica*/*E. dispar* complex, which may include other species such as *Entamoeba moshkovskii* and *Entamoeba hartmanni* [9].

Among the enteric parasites, soil-transmitted helminths (STHs) are targeted by preventive chemoprophylaxis with a periodic 400 mg albendazole dose [10]. This may have shaped the etiological profile of parasitic intestinal infections towards a higher frequency of protozoa detection [11]. There are no antiprotozoal drugs compatible with chemoprophylaxis, and treatment must be individualized with the dose being adjusted for body weight [12–14]. The most available drug, metronidazole, should be taken for five days at eight-hour intervals [15]. A single dose drug option, secnidazole also requires individualized body weight prescription [16]. Nitazoxanide, a more recently proposed option, is not economically viable for use on a community scale [17]. The present study aims to characterize the prevalence, distribution, and etiological profile of intestinal parasitism in children living in periurban areas with poor sanitation in the state of Rio de Janeiro, Brazil.

## 2. Materials and Methods

### 2.1. Description of the Studied Area

**Figure 1:**
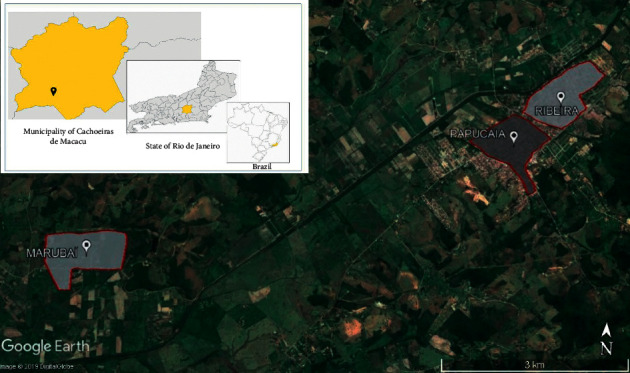
Basic rocket ship design. The rocket ship is propelled with three thrusters and features a single viewing window. The nose cone is detachable upon impact.

The study was carried out in Papucaia, Ribeira, and Marubaí in the municipality of Cachoeiras de Macacu, Rio de Janeiro (Figure 1).

Papucaia and Ribeira are periurban districts, in which almost 3,000 families (approximately 17,000 inhabitants) live. In general, in the communities of Papucaia and Ribeira, treated piped water is supplied to households. In homes, water is stored in tanks and consumed directly, without any further treatment. There is a sewage system, and evacuation is practiced in latrines inside the houses. Solid excreta, however, is discharged without treatment into water bodies such as rivers. In Marubaí, the drinking water comes from artesian wells.

### 2.2. Study Design and Sampling Strategy

A community-based cross-sectional survey was carried out in 2018 and included 479 children aged 0–15 years (209 in Papucaia, 180 in Ribeira, and 90 in Marubaí). Sampling included 36.7% of children living in Papucaia, 30.2% of those in Ribeira, and 70.8% of those in Marubaí. With the sample size reached, we had an 80% confidence level to identify prevalence rates with an expected frequency of 20% and a margin of error of 2%. The researchers asked questions in a standardized questionnaire to obtain sociodemographic and sanitation data. Per capita household income was calculated by summing the income of all household members and dividing by the number of residents. Children were classified as poor when living in families with incomes below R$178 (equivalent to approximately 44 USD) and 43.2% of children were classified as poor.

### 2.3. Ethical Approval

The study was previously approved by the Research Ethics Committee of Instituto Oswaldo Cruz/Fiocruz, license number CAAE: 12125713.5.0000.5248.

### 2.4. Parasitological Examinations

Three faecal samples collected on successive days were examined per child. Samples were processed through Ritchie's modified ethyl acetate sedimentation technique [18].

### 2.5. Statistical Analyses

Data were presented descriptively, and statistical analyses were performed with SPSS® (IBM Corp., Armonk, NY, USA) as prevalence rates of different parasite species in distinct sociodemographic categories. Prevalence ratios and respective 95% CIs were calculated. The statistical significance of the associations was assessed by Fisher's exact test, with a significance threshold of *p* < 0.05.

## 3. Results

**Figure 2:**
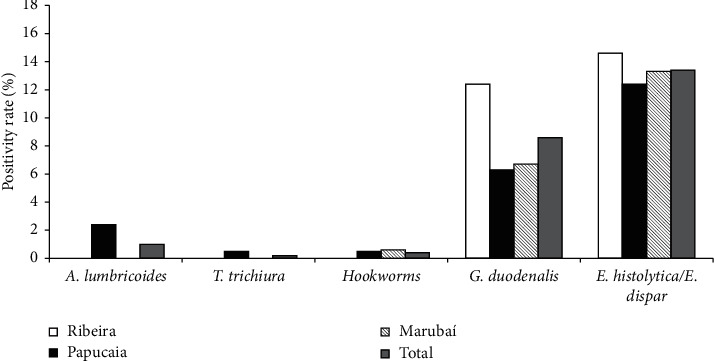
Prevalence rates of infections by different intestinal parasites in children aged 0–14 years in three communities of Cachoeiras de Macacu, Rio de Janeiro, 2018.

Among the children included in the study, the prevalence of infection by any organism was 19.4% (93/479).  Figure 2 presents the positivity rates for distinct pathogenic organisms by community. Eight children (1.7%) were positive for STH (*A. lumbricoides* (*n* = 5), hookworms (*n* = 2), or *T. trichiura* (*n* = 1)).

The positivity rate for any pathogenic protozoan was 17.9%.  Table 1 shows the distribution of infections with *Giardia duodenalis* and *E. histolytica*/*E. dispar*. Prevalence of infection with *G. duodenalis* and *E. histolytica*/*E. dispar* was 8.6% (*n* = 41) and 13.4% (*n* = 64), respectively, in all localities. Infection with *G. duodenalis* was significantly more frequent among children living in poor families. This difference was also significant for infection with any pathogenic parasite. In addition, people residing in houses with more than four inhabitants showed significantly higher positivity for infections with *G. duodenalis* and *E. histolytica*/*E. dispar* (Table 1). Prevalence rates of infection with pathogenic protozoa were significantly lower among children aged up to two years. There were no significant differences in the positivity rates across the three studied locations (Marubaí, Papucaia, and Ribeira).

In relation to positivity for nonpathogenic protozoa, the prevalence rates were 9.2% (*n* = 44) for *Endolimax nana*, 1.3% (*n* = 6) for *Iodamoeba butschlii*, and 5.8% (*n* = 28) for *Entamoeba coli*.

Among the 93 positive samples for any pathogenic organism, 86 had pathogenic protozoa, eight had STH, and one had both. Anthelmintics were reported to be used in 136 children (27.6%) during the period from one to six months prior to faecal collection. Information on the presence of diarrhoea was available for 439 (91.7%) of 479 children. It was observed that 23 (5.2%) presented diarrhoea at some point previous to faecal sample collection. Of these, 16 reported symptoms within 15 days before collection, two had diarrhoea within 15 to 30 days prior to collection, and five reported it more than one month before. There were no significant differences in positivity rates for different parasites among children who reported and did not report diarrhoea (37/416 (8.9%) vs. 0/23 (0%); *p*=0.243 for *G. duodenalis* and 60/416 (14.4%) vs. 2/23 (8.7%); *p*=0.757 for *E. histolytica*/*E. dispar*.

## 4. Discussion

This study revealed the predominance of protozoa among organisms that parasitize the digestive tract of children in a periurban area of low socioeconomic status in the state of Rio de Janeiro, Brazil. Similar results were found in other studies conducted in Rio de Janeiro [19–21]. Regarding infections in different age groups, significantly higher positivity rates in children aged from three to six were observed, suggesting a greater exposure at these ages.

The control strategies for intestinal parasites aim for the elimination of STHs, and there is a trend of reduction in their prevalence [22]. In this study, almost a third of children had used drugs such as albendazole and mebendazole during the six months before faecal examination. Despite this, studies have demonstrated that in certain regions of Brazil—mainly in rural areas—some STH foci persist [23, 24].

The administration of anthelmintic drugs without appropriate diagnostic tests has made it difficult to diagnose infection with protozoa, making giardiasis and amoebiasis underdiagnosed diseases. Techniques capable of detecting intestinal protozoan infections have not been performed in public health laboratories. More recently, enzyme immunoassay has replaced microscopic examination in most clinical laboratories [25]. However, the cost of these tests does not yet allow their use for large-scale diagnosis in primary health care.

In this study, although a few children reported diarrhoea in the period before stool collection, no diarrhoeal samples were found, indicating chronic and apparently asymptomatic infections. Although recognized as a cause of epidemic water-borne diarrhoeal disease and traveller's diarrhoea in developed countries, the role of *G. duodenalis* as an etiological agent of diarrhoeal diseases in developing countries is less certain [26, 27]. Interestingly, recent studies failed to characterize *G. duodenalis* as a pathogen associated with diarrhoeal disease in children in developing countries. Instead, giardiasis presents as chronic and apparently asymptomatic or causes mild illness of the small intestine, associated with chronic nutrient spoliation, and deficits in physical development due to interference with absorptive function [28, 29].

Infection by *E. histolytica*/*E. dispar* was observed in the three studied locations in all age groups. The positivity rate was akin to that described in similar studies conducted in other regions. Among the positive children, none had symptoms compatible with invasive conditions that could indicate the presence of amoebic dysentery. It should be in mind that the clinical manifestations of *E. histolytica*/*E. dispar* infection have variable symptoms, from subclinical colonization to severe invasive conditions. It is not possible to differentiate, under light microscopy, the species of the *E. histolytica*/*E. dispar* complex. Additionally, it is possible that, in the studied communities, low virulence strains predominate, as observed in several countries. As faecal-borne diseases, giardiasis and amoebiasis are sensitive to sanitation conditions and the supply of drinking water. Thus, they have a strong social determination and are associated with poverty. Household crowding, poor sanitation, and water supply have been associated with intestinal protozoa infection [30, 31].

## 5. Conclusions

This study illustrates the changing etiological profile of intestinal parasitism in periurban areas in Brazil and points to the need for the improvement of control strategies, which should include enhancements in sanitation. Laboratory diagnosis of protozoan enteric infections and effective drugs for their treatment are unmet goals in the primary health care system. Therefore, giardiasis and amebiasis are neglected conditions.

## Figures and Tables

**Table 1:** Distribution of *Giardia duodenalis* and *E. histolytica*/*E. dispar* infections in Cachoeiras de Macacu, RJ, 2018.

Locality	*Giardia duodenalis*			*E. histolytica*/*E. dispar*			Any pathogenic protozoa		
	Prevalence	Prevalence ratio^*∗∗*^	*p* value	Prevalence	Prevalence ratio^*∗∗*^	*p* value	Prevalence	Prevalence ratio^*∗∗*^	*p* value
Papucaia	13/209 (6.2%)	1		26/209 (12.4%)	1		31/209 (14.8%)	1	
Marubaí	6/90 (6.7%)	1.07 (0.42–2.73)	1	12/90 (13.3%)	1.07 (0.56–2.02)	0.850	15/90 (16.7%)	1.12 (0.63–1.97)	0.727
Ribeira	22/180 (12.2%)	1.96 (1.01–3.78)	0.049	26/180 (14.4%)	1.16 (0.70–1.92)	0.654	40/180 (22.2%)	1.49 (0.97–2.29)	0.066
Age group (years)									
0–2	2/94 (2.1%)	1		4/94 (4.3%)	1		6/94 (6.4%)	1	
3–6	17/154 (11%)	5.18 (1.22–21.95)	0.012	23/154 (14.9%)	3.50 (1.25–9.83)	0.010	32/154 (20.8%)	3.25 (1.41–7.49)	0.001
7–15	22/231 (9.5%)	4.47 (1.07–18.65)	0.019	37/231 (16%)	3.76 (1.38–10.26)	0.002	48/231 (20.8%)	3.25 (1.44–7.34)	<0.001
Income per capita per month^*∗*^ (USD)									
<44 USD^1^	24/187 (12.8%)	2.18 (1.19–3.99)	0.011	31/187 (16.6%)	1.45 (0.91–2.30)	0.127	43/187 (23%)	1.56 (1.06–2.30)	0.026
≥44 USD^2^	16/272 (5.9%)	1		31/272 (11.4%)	1		40/272 (14.7%)	1	
Number of persons in the household									
≤4	16/311 (5.1%)	1		30/311 (9.6%)	1		41/311 (13.2%)	1	
>4	22/138 (15.9%)	3.09 (1.68–5.71)	<0.001	32/138 (23.2%)	2.40 (1.52–3.79)	<0.001	40/138 (29%)	2.19 (1.49–3.23)	<0.001
Gender									
Male	21/250 (8.4%)	1		39/250 (15.6%)	1		49/250 (19.6%)	1	
Female	20/229 (8.7)	1.03 (0.57–1.86)	1	25/229 (10.9%)	0.69 (0.43–1.11)	0.141	37/229 (16.2%)	0.82 (0.55–1.21)	0.342

^*∗*^USD 1 = BRL 4. ^*∗∗*^95% CI. ^1^Poverty. ^2^Not poverty.

## Data Availability

The data used to support this study are available from the corresponding author upon request. The data are not publicly available because they contain information that could compromise the privacy of research participants.
